# Supplementary material for: The Salmonella pathogenicity island 1 injectisome reprograms host cell translation to evade the inflammatory response
Source: Nat Commun. 2025 Nov 4;16:9742. doi: 10.1038/s41467-025-64744-w (PMC12586433; doi:10.1038/s41467-025-64744-w)
Supplement: Supplementary file 1 — Supplementary Information [file 41467_2025_64744_MOESM1_ESM.pdf]

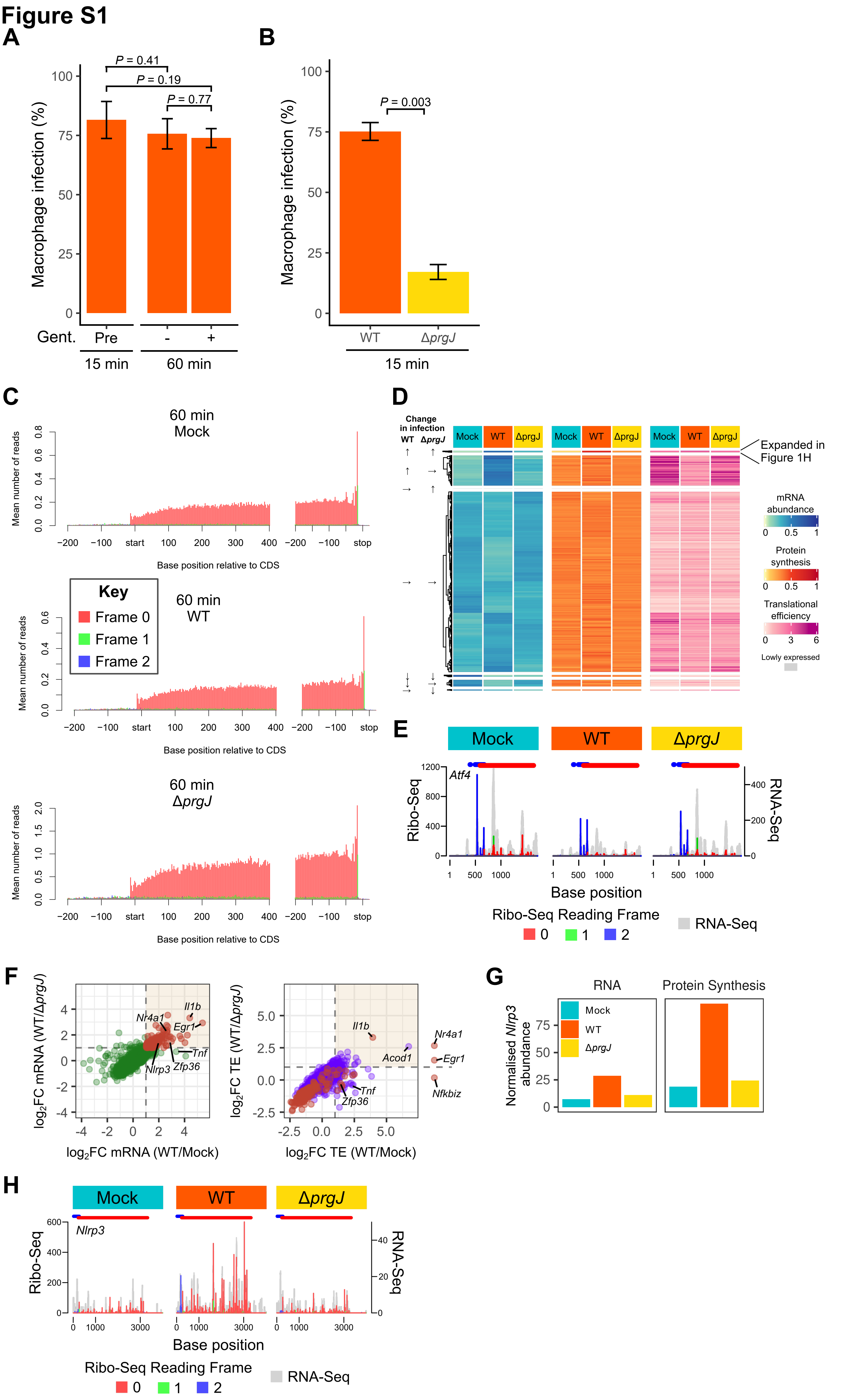

Fig S1: (**A**) Percentage of macrophages infected by WT *Salmonella* determined by microscopy at 15 and 60 min, with and without addition of gentamicin (Gent) at 15 min. Significance determined by two-sided Student's t-test; two independent experiments, ~100 cells per condition, at least 5 technical replicates per condition. (**B**) Percentage of macrophages infected by WT or Δ*prgJ Salmonella* determined by microscopy at 15 min. Significance determined by two-sided Student's t-test; n=2. (**C**) Meta-gene translatome from ribosome profiling of *Salmonella* infected macrophages at 60 min. Histograms of RPF 5′ ends relative to start and stop codons colored by their reading frame relative to the coding sequence. (**D**) Heatmap showing mRNA abundance, protein synthesis and TE of all genes in **Fig 1G**. Genes are ordered by hierarchical clustering of mRNA abundance across all conditions (left). Arrows indicate direction of differential transcript abundance (log2FC ±1) in the indicated infection vs mock. (**E**) Normalized Ribo-Seq read count and RNA-Seq transcript coverage of *Atf4*. Ribo-Seq reads are represented by their P site position and colored by their reading frame relative to the start codon of the main *Atf3* coding sequence. Open reading frames are represented by bars above each plot. (**F**) Comparison of changes in mRNA abundance (left) and TE (right) on infection with WT over mock infection or Δ*prgJ Salmonella*. Genes upregulated transcriptionally in both (log2FC > 1) are shown in red. *Egr1*, *Nr4a1* and *Nfkbiz* are plotted separately due to low abundance in mock infection precluding accurate calculation of TE, and as such TE fold change in WT *Salmonella* over mock infection. (**G**) Normalized mRNA abundance and protein synthesis of *Nlrp3* at 60 min post-infection. (**H**) Normalized Ribo-Seq read count and RNA-Seq transcript coverage of *Nlrp3* as in E. The *Nlrp3* uORF can be readily seen as reads in a different reading frame within the 5' UTR.

Figure S2

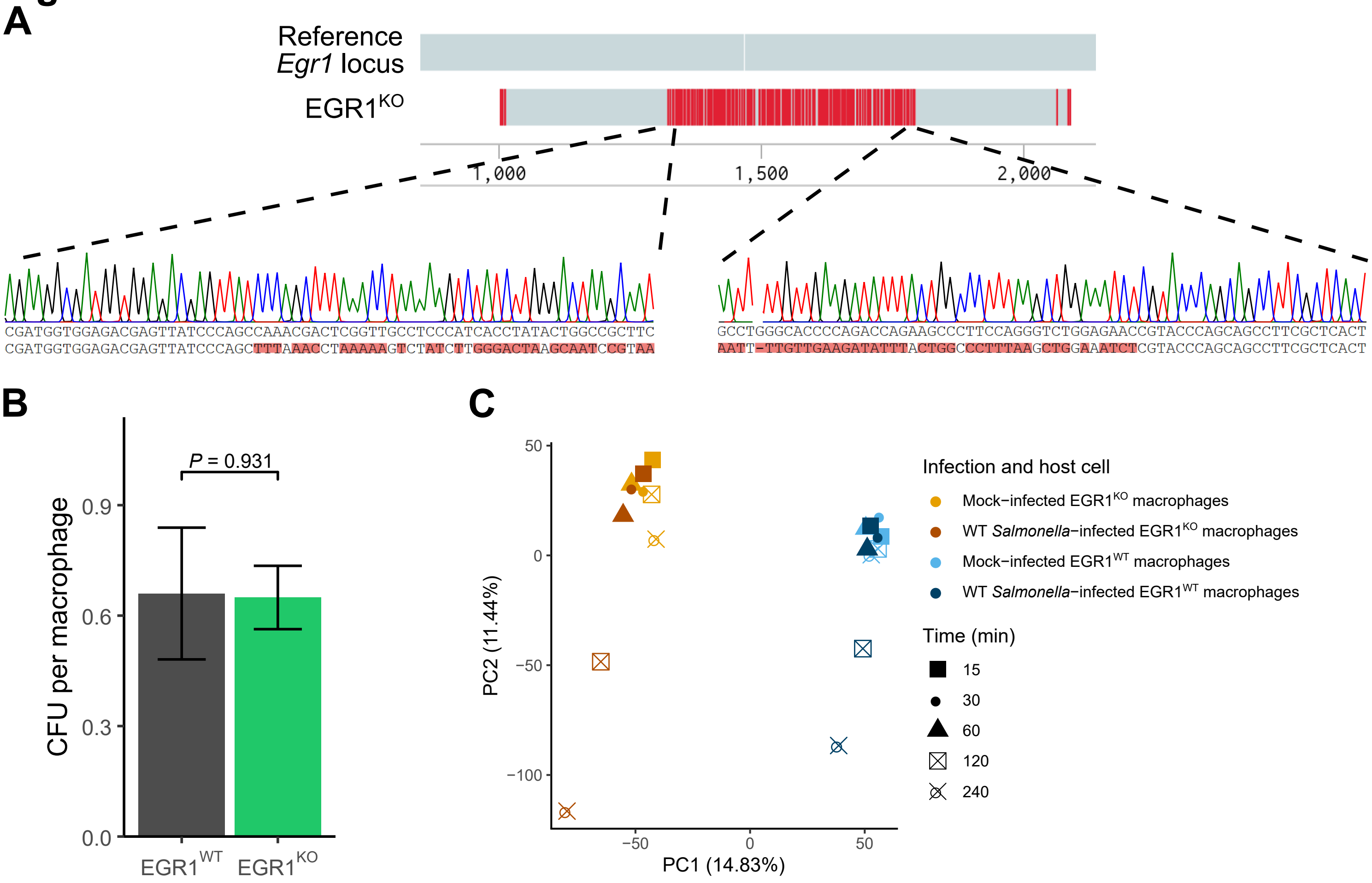

Fig S2: (**A**) Example section of the *Egr1* coding sequence with an alignment from targeted sequencing of the *Egr1* locus in the EGR1 knockout (KO) macrophages. Mismatched bases and deletions are highlighted in red. (**B**) *Salmonella* colony forming units (CFU) recovered 75 min post WT *Salmonella* infection of EGR1 KO or WT control macrophages. Significance determined by two-sided Student's t-test; *n*=3. (**C**) Principal component analysis of the transcriptomes of WT *Salmonella* infected or mock infected EGR1 KO or WT control macrophages over an infection time-course of 240 min.

Figure S3

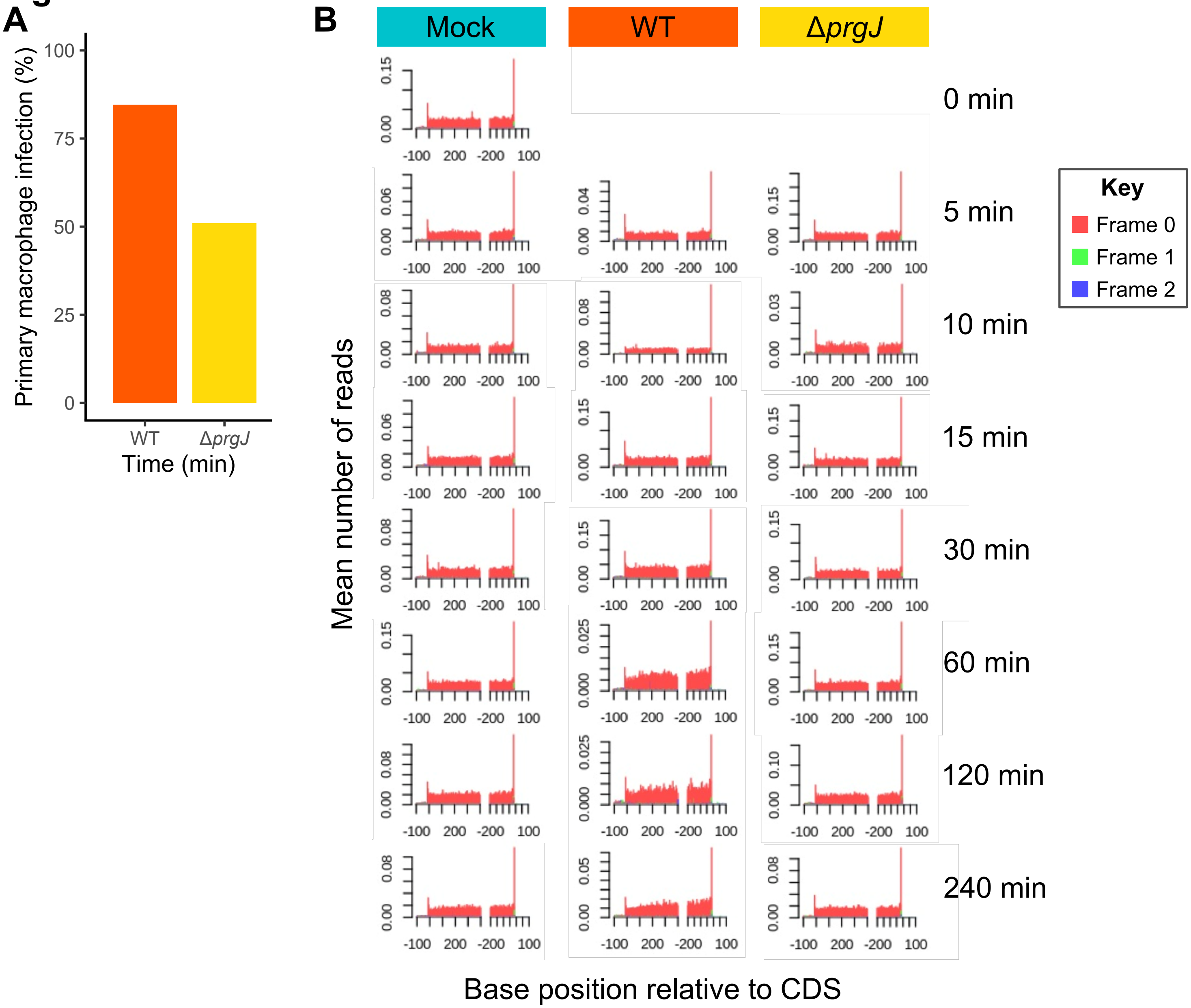

Fig S3: (**A**) Percentage of primary macrophages infected by WT or Δ*prgJ Salmonella* determined by microscopy at 15 min post infection. (**B**) Meta-gene translatome from ribosome profiling across a *Salmonella* primary bone marrow derived macrophage infection time-course. Histograms of RPF 5′ ends relative to start and stop codons colored by their reading frame relative to the coding sequence.

Figure S4

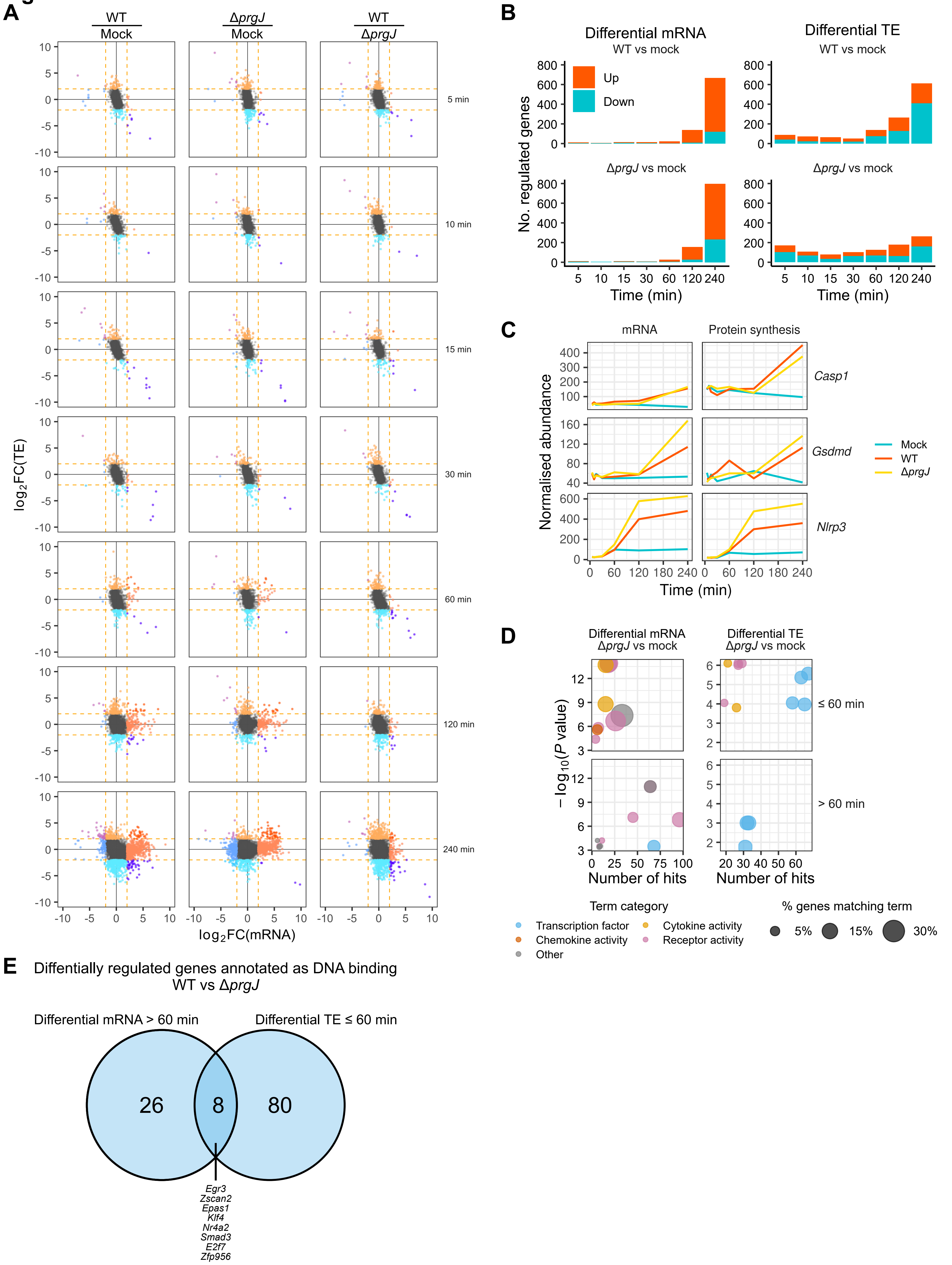

Fig S4: (**A**) log2FC TE vs log2FC mRNA abundance between the indicated infections, across the time-course. Dashed orange lines show the $\log_2$FC cutoffs (±2) used to select differentially expressed genes; genes that pass these thresholds are colored. (**B**) Number of genes differentially expressed between WT vs mock and Δ*prgJ* vs mock infection at both the TE (right) and mRNA (left) levels. (**C**) Expression of genes encoding components of the inflammasome [74] that are upregulated over the infection time-course. (**D**) Top 10 enriched GO molecular function terms in differentially expressed genes at both the TE (right) and mRNA (left) levels. Genes were split by when they were differentially expressed: at or before 60 min, and after 60 min post infection. (**E**) Overlap of genes with DNA binding and transcription related annotations that are differentially regulated on the translational level at or before 60 min or the transcriptional level after 60 min in the comparison of WT vs Δ*prgJ* infection.

Figure S5

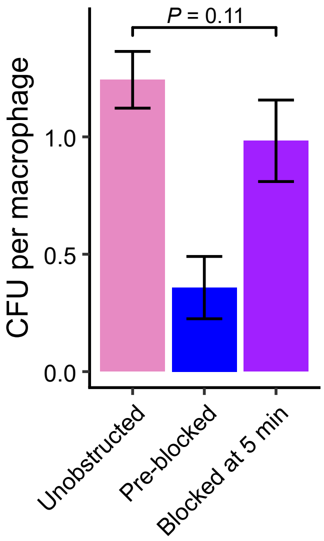

Fig S5: *Salmonella* colony forming units (CFU) recovered from macrophages 75 min post infection with blockage of the SPI-1 injectisome induced as indicated.
