# Supplementary material for: The Salmonella pathogenicity island 1 injectisome reprograms host cell translation to evade the inflammatory response
Source: Nat Commun. 2025 Nov 4;16:9742. doi: 10.1038/s41467-025-64744-w (PMC12586433; doi:10.1038/s41467-025-64744-w)
Supplement: Supplementary file 2 — Description of Additional Supplementary Files [file 41467_2025_64744_MOESM2_ESM.pdf]

Title: Supplementary Data 1

Description: List of host genes transcriptionally upregulated in WT Salmonella infected iBMDMs on EGR1 KO. Gene order is the same presented in the heatmap Fig 3H

Title: Supplementary Data 2

Description: G:profiler molecular function and biological process enrichment of genes from transcriptionally upregulated in WT Salmonella infected iBMDMs on EGR1 KO. P values were determined with Fisher's one-tailed test with Benjamini-Hochberg correction for multiple comparison.

Title: Supplementary Data 3

Description: G:profiler molecular function enrichment of genes with differential TE and mRNA abundance during infection of primary BMDMs. P values were determined with Fisher's one-tailed test with Benjamini-Hochberg correction for multiple comparison.

Title: Supplementary Data 4

Description: qPCR primer sequences

Title: Supplementary Data 5

Description: CRISPR gRNA sequences for EGR1 KO

Title: Supplementary Data 6

Description: Normalised Ribo- and RNA-Seq counts from Salmonella in broth or macrophages for selected SPI-1, SPI-2, flagella or intramacrophage genes detected in samples from infected macrophages

Title: Supplementary Data 7

Description: Statistical tests displayed in figures
