# Supplementary material for: The Salmonella pathogenicity island 1 injectisome reprograms host cell translation to evade the inflammatory response
Source: Nat Commun. 2025 Nov 4;16:9742. doi: 10.1038/s41467-025-64744-w (PMC12586433; doi:10.1038/s41467-025-64744-w)
Supplement: Supplementary file 4 — Reporting Summary [file 41467_2025_64744_MOESM4_ESM.pdf]

nature portfolio

Corresponding author(s): Betty Y.W. Chung and Clare E. Bryant

Last updated by author(s): Jul 10, 2025

Reporting Summary

Nature Portfolio wishes to improve the reproducibility of the work that we publish. This form provides structure for consistency and transparency in reporting. For further information on Nature Portfolio policies, see our Editorial Policies and the Editorial Policy Checklist.

Statistics

For all statistical analyses, confirm that the following items are present in the figure legend, table legend, main text, or Methods section.

n/a	Confirmed	
☐	☒	The exact sample size (n) for each experimental group/condition, given as a discrete number and unit of measurement
☐	☒	A statement on whether measurements were taken from distinct samples or whether the same sample was measured repeatedly
☐	☒	The statistical test(s) used AND whether they are one- or two-sided<br>*Only common tests should be described solely by name; describe more complex techniques in the Methods section.*
☐	☒	A description of all covariates tested
☐	☒	A description of any assumptions or corrections, such as tests of normality and adjustment for multiple comparisons
☐	☒	A full description of the statistical parameters including central tendency (e.g. means) or other basic estimates (e.g. regression coefficient) AND variation (e.g. standard deviation) or associated estimates of uncertainty (e.g. confidence intervals)
☐	☒	For null hypothesis testing, the test statistic (e.g. F, t, r) with confidence intervals, effect sizes, degrees of freedom and P value noted<br>*Give P values as exact values whenever suitable.*
☒	☐	For Bayesian analysis, information on the choice of priors and Markov chain Monte Carlo settings
☒	☐	For hierarchical and complex designs, identification of the appropriate level for tests and full reporting of outcomes
☒	☐	Estimates of effect sizes (e.g. Cohen's d, Pearson's r), indicating how they were calculated

Our web collection on statistics for biologists contains articles on many of the points above.

Software and code

Policy information about availability of computer code

Data collection	No software was used.
Data analysis	R (3.6.0); RiboSeqR (1.20.0); edgeR (3.28.1); xtail (1.1.5); ComplexHeatmap (2.2.0); gprofiler2 (0.2.1); bowtie (1.2.3); FASTX-Toolkit (0.0.13); htseq (0.12.4); STAR (2.7.4a); QuantStudio Design and Analysis Software (Applied Biosystems, 2.8); ImageStudio Lite (Licor, 5.2)

For manuscripts utilizing custom algorithms or software that are central to the research but not yet described in published literature, software must be made available to editors and reviewers. We strongly encourage code deposition in a community repository (e.g. GitHub). See the Nature Portfolio guidelines for submitting code & software for further information.

Data

Policy information about availability of data

All manuscripts must include a data availability statement. This statement should provide the following information, where applicable:

- Accession codes, unique identifiers, or web links for publicly available datasets
- A description of any restrictions on data availability
- For clinical datasets or third party data, please ensure that the statement adheres to our policy

Raw and processed ribosome profiling and RNA sequencing data are available from the European Nucleotide Archive under study accessions ERP149959 [https://www.ebi.ac.uk/ena/browser/view/ERP149959] and ERP179251 [https://www.ebi.ac.uk/ena/browser/view/ERP179251] or can be found in the supplementary tables. Source data are provided with this paper.

Research involving human participants, their data, or biological material

Policy information about studies with human participants or human data. See also policy information about sex, gender (identity/presentation), and sexual orientation and race, ethnicity and racism.

Reporting on sex and gender	NA
Reporting on race, ethnicity, or other socially relevant groupings	NA
Population characteristics	NA
Recruitment	NA
Ethics oversight	NA

Note that full information on the approval of the study protocol must also be provided in the manuscript.

Field-specific reporting

Please select the one below that is the best fit for your research. If you are not sure, read the appropriate sections before making your selection.

☒ Life sciences ☐ Behavioural & social sciences ☐ Ecological, evolutionary & environmental sciences

For a reference copy of the document with all sections, see nature.com/documents/nr-reporting-summary-flat.pdf

Life sciences study design

All studies must disclose on these points even when the disclosure is negative.

Sample size	No sample size calculation was performed. The number of mice used was minimised, with futher validation of findings in cell lines.
Data exclusions	Genes with low read counts (<10 reads) were excluded from analysis as detailed in the methods.
Replication	Experiments were replicated as detailed in the methods and in figure legends. No replicates were excluded from analysis.
Randomization	Experiments utilized cells from the same source for testing of control and experimental variables and so randomization was not necessary.
Blinding	Blinding investigators is was not necessary as experimental outputs were objectively measured using standardized assays. All sequencing data was analyzed with the same computational pipeline.

Reporting for specific materials, systems and methods

We require information from authors about some types of materials, experimental systems and methods used in many studies. Here, indicate whether each material, system or method listed is relevant to your study. If you are not sure if a list item applies to your research, read the appropriate section before selecting a response.

Materials & experimental systems

n/a	Involved in the study	
☐	☒	Antibodies
☐	☒	Eukaryotic cell lines
☒	☐	Palaeontology and archaeology
☐	☒	Animals and other organisms
☒	☐	Clinical data
☒	☐	Dual use research of concern
☒	☐	Plants

Methods

n/a	Involved in the study	
☒	☐	ChIP-seq
☒	☐	Flow cytometry
☒	☐	MRI-based neuroimaging

Antibodies

Antibodies used	rabbit anti-EGR1 (Cell Signaling, #44D5, 1:1000); mouse anti-GAPDH (Sigma Aldrich, #G8795, 1:2000); mouse anti-SipC (tgcBIOMICS, #tgc-a201-1, 1:1000); rabbit anti-GroEL (Abcam, #Ab90522, 1:80000); anti-Myc-HRP conjugate (Cell Signaling, #2040, 1:1000). Anti-rabbit conjugated to HRP (Cell Signaling, #7074, 1:20000); anti-mouse conjugated to IRDye 800 CW (Licor, #926-32210, 1:5000); anti-

mouse IgG antibody conjugated to HRP (Promega, #W4021, 1:20000); anti-rabbit IgG antibody conjugated to HRP (Promega, #W4011, 1:20000); goat anti-Salmonella CSA-1 (Insight Biotechnology, #01-91-99, 1:200); anti-goat IgG conjugated to Alexa488 (Abcam, #ab150077, 1:200)

Validation	Anti-EGR1 and anti-GAPDH validated by manufacturer by western blot of mouse protein. Anti-SipC and anti-GroEL validated by western blot on Salmonella protein in this study (Fig 4B). Anti-Myc-HRP conjugate validated by western blot on protein from Salmonella expressing Myc tagged SptP-GFP in this study (Fig 4B). All proteins were of expected size and only present in expected conditions. Anti-Salmonella CSA-1 was validated by IF microscopy (Fig 1A).

Eukaryotic cell lines

Policy information about cell lines and Sex and Gender in Research

Cell line source(s)	Immortalized bone marrow derived macrophages (iBMDM) derived from male 8-week old C57BL/6 mice.
Authentication	Confirmed by microscopy and stereotypical phenotypic responses (Fig. 1A-D)
Mycoplasma contamination	All cell lines were routinely tested to ensure they were free of mycoplasma contamination.
Commonly misidentified lines (See ICLAC register)	NA

Animals and other research organisms

Policy information about studies involving animals; ARRIVE guidelines recommended for reporting animal research, and Sex and Gender in Research

Laboratory animals	BMDMs were generated from male 8-week old C57BL/6 mice.
Wild animals	NA
Reporting on sex	Only cells derived from male mice were used in this study. Sexing was performed by trained and experienced animal handlers.
Field-collected samples	NA
Ethics oversight	All mice were maintained in a specific pathogen-free facility according to the Animals Scientific Procedures outlined by the UK Home Office regulations. All work involving live animals complied with the University of Cambridge Ethics Committee regulations and was performed under the Home Office Project License numbers 80/2572 and P48B8DA35.

Note that full information on the approval of the study protocol must also be provided in the manuscript.

Plants

Seed stocks	NA
Novel plant genotypes	NA
Authentication	NA
