# Supplementary figures and images for: The Salmonella pathogenicity island 1 injectisome reprograms host cell translation to evade the inflammatory response
Source: Nat Commun. 2025 Nov 4;16:9742. doi: 10.1038/s41467-025-64744-w (PMC12586433; doi:10.1038/s41467-025-64744-w)

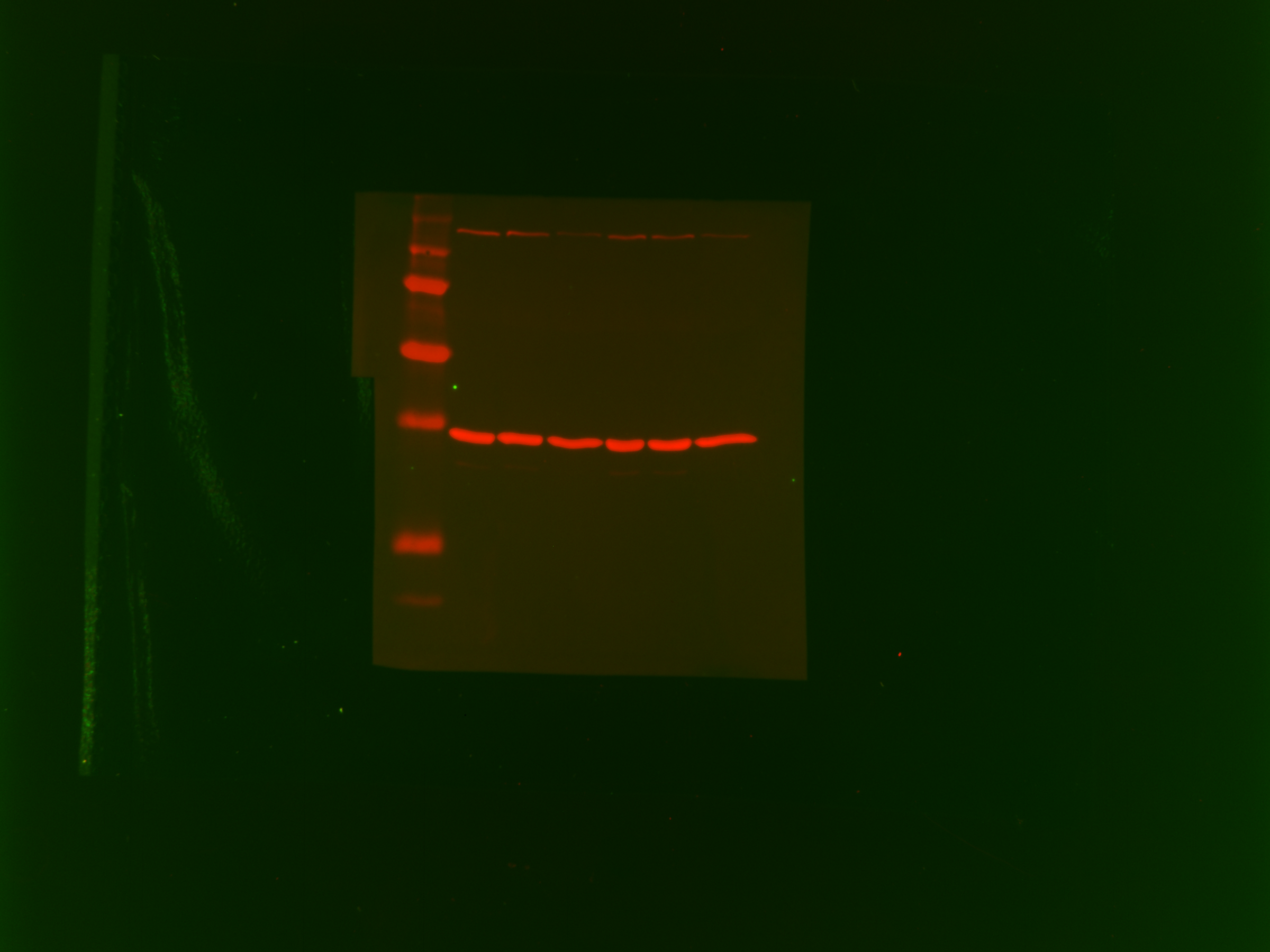

Supplement: Supplementary file 6 — Source data [file 41467_2025_64744_MOESM6_ESM.zip › 2C Membrane2 EGR1 NIR.tif]

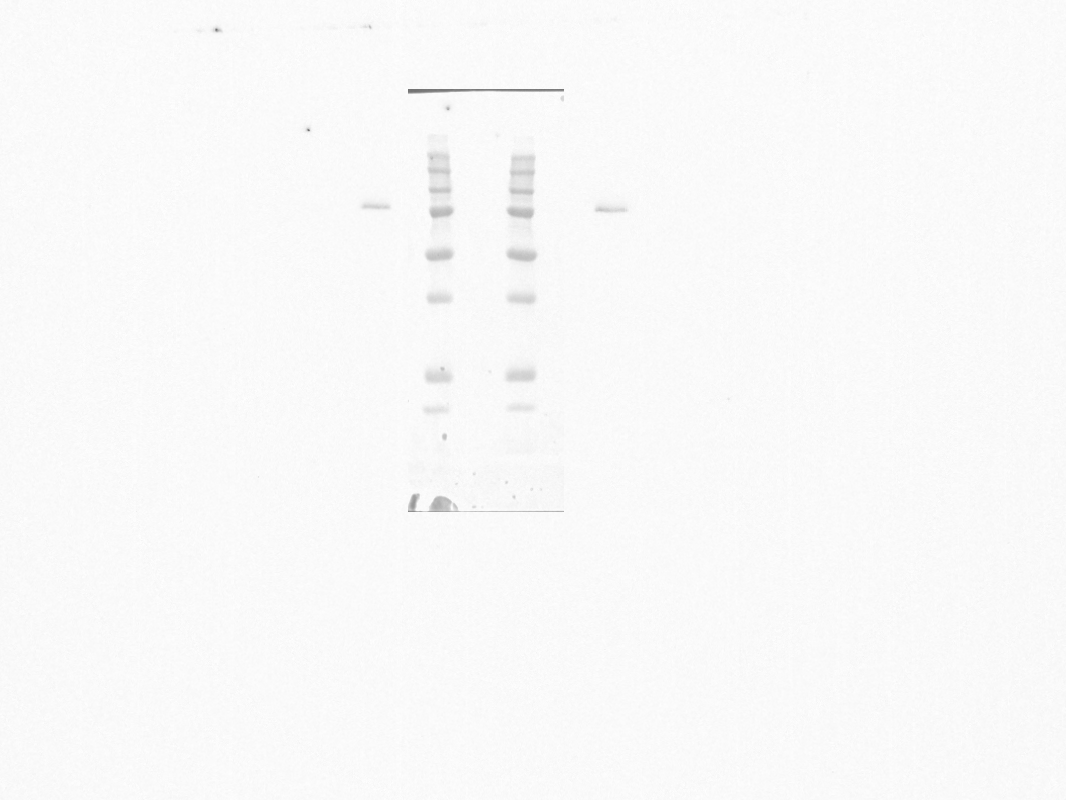

Supplement: Supplementary file 6 — Source data [file 41467_2025_64744_MOESM6_ESM.zip › 2E EGR1 KO chemi marker.tif]

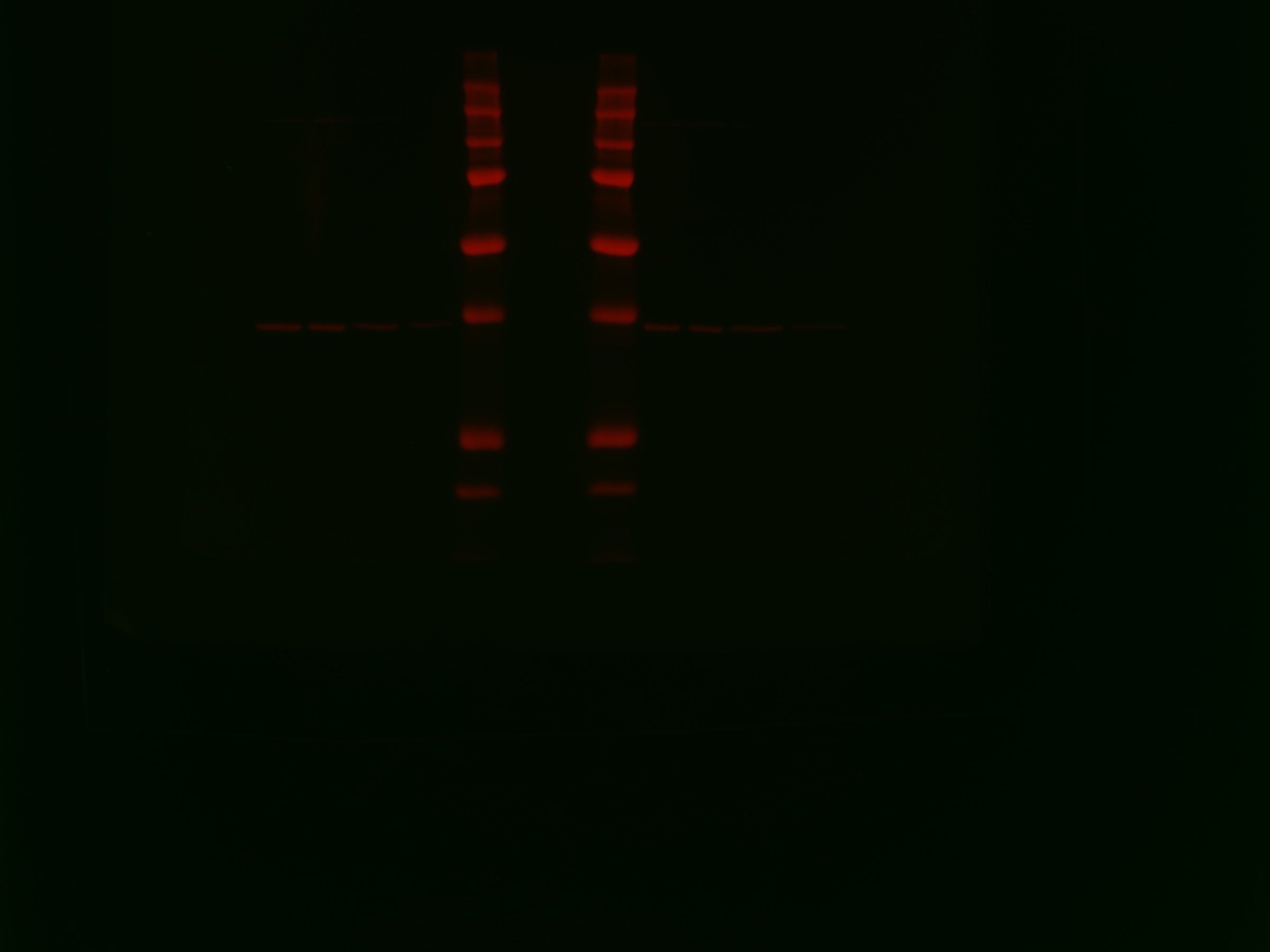

Supplement: Supplementary file 6 — Source data [file 41467_2025_64744_MOESM6_ESM.zip › 2E EGR1 KO NIR.tif]

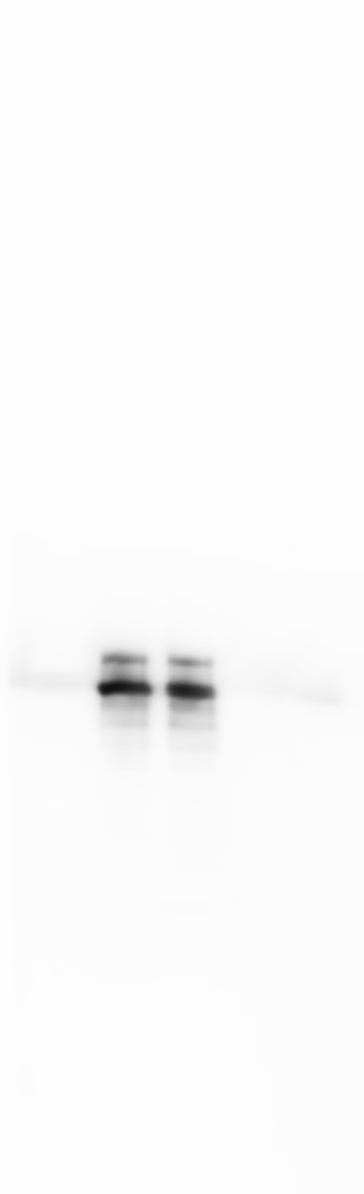

Supplement: Supplementary file 6 — Source data [file 41467_2025_64744_MOESM6_ESM.zip › 4B GroEL.png]

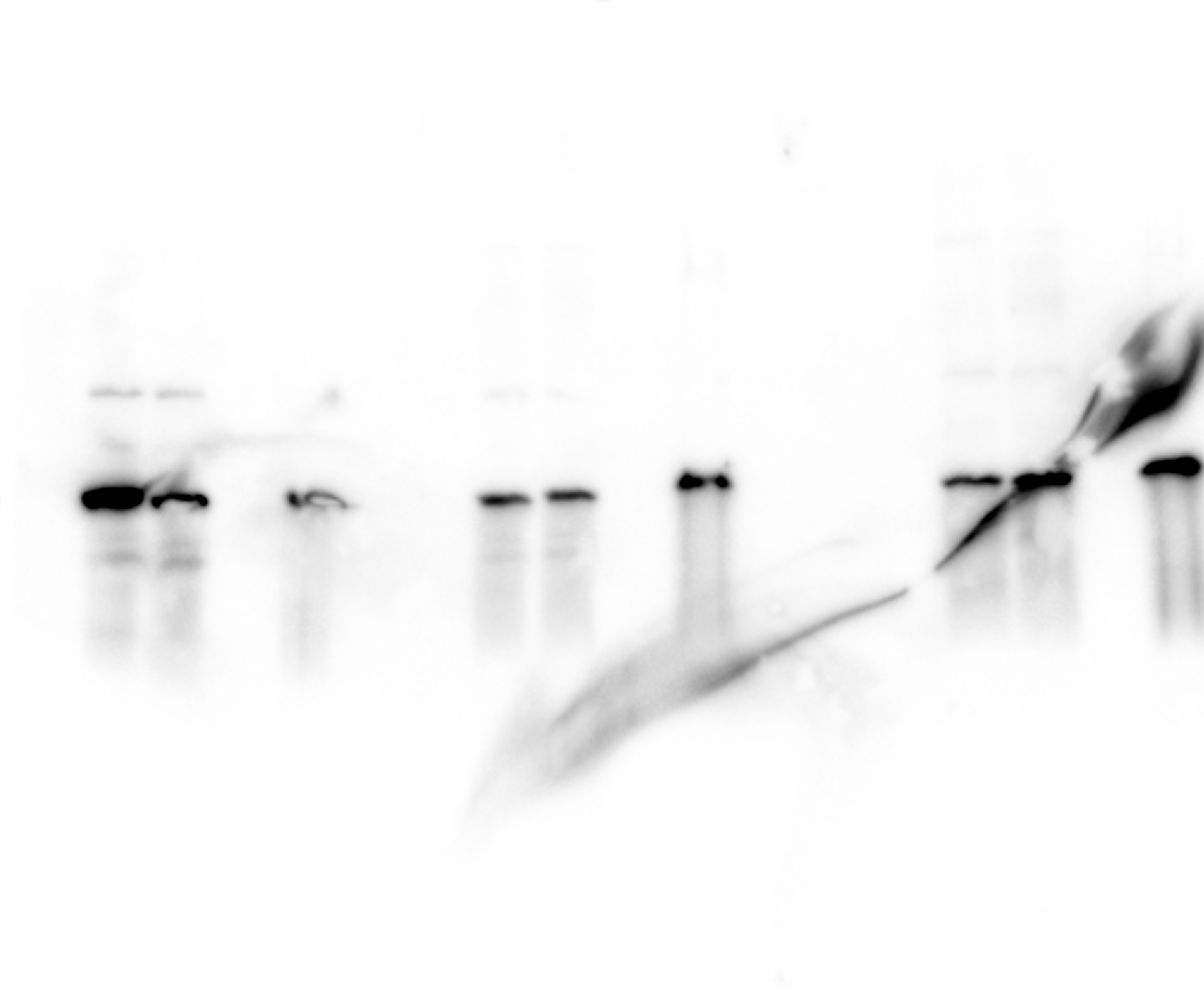

Supplement: Supplementary file 6 — Source data [file 41467_2025_64744_MOESM6_ESM.zip › 4B SipC.png]

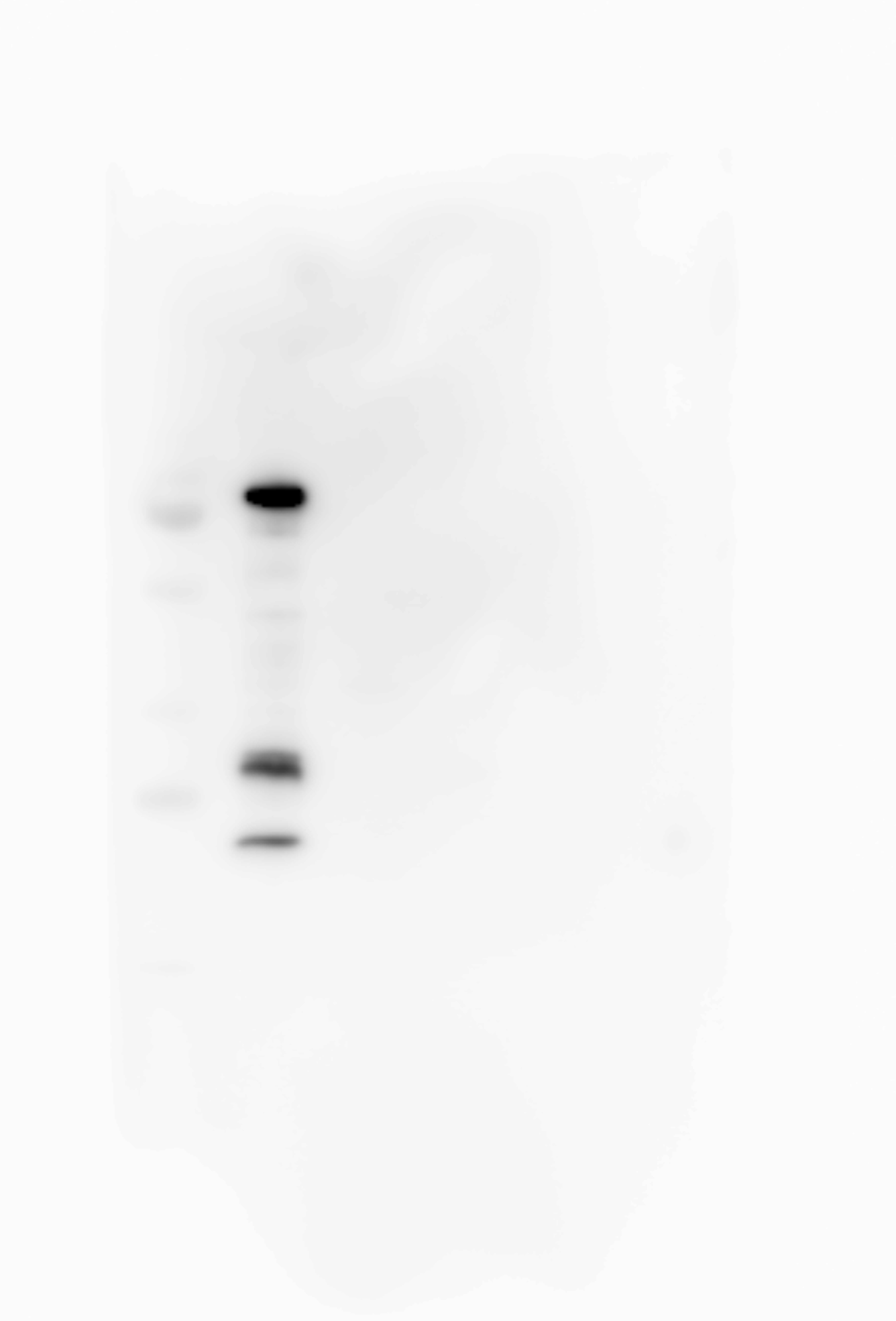

Supplement: Supplementary file 6 — Source data [file 41467_2025_64744_MOESM6_ESM.zip › 4B SptP_GFP.png]

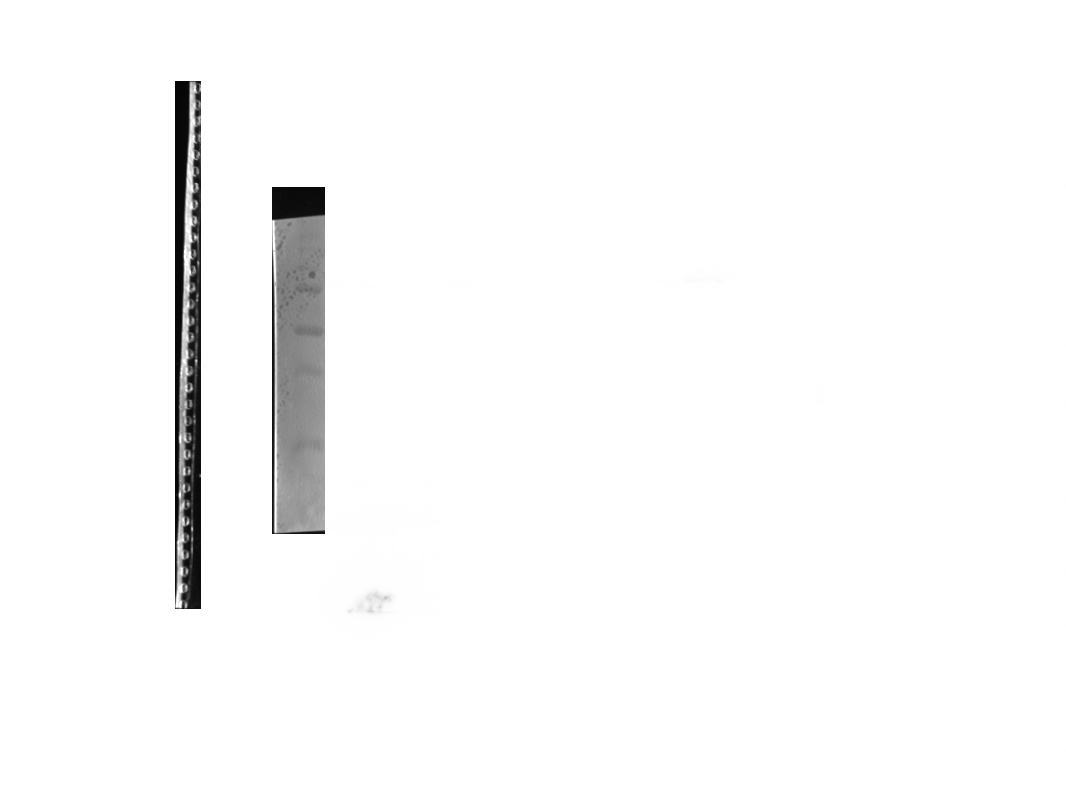

Supplement: Supplementary file 6 — Source data [file 41467_2025_64744_MOESM6_ESM.zip › 2C Membrane1 EGR1 Chemi Normal.tif]

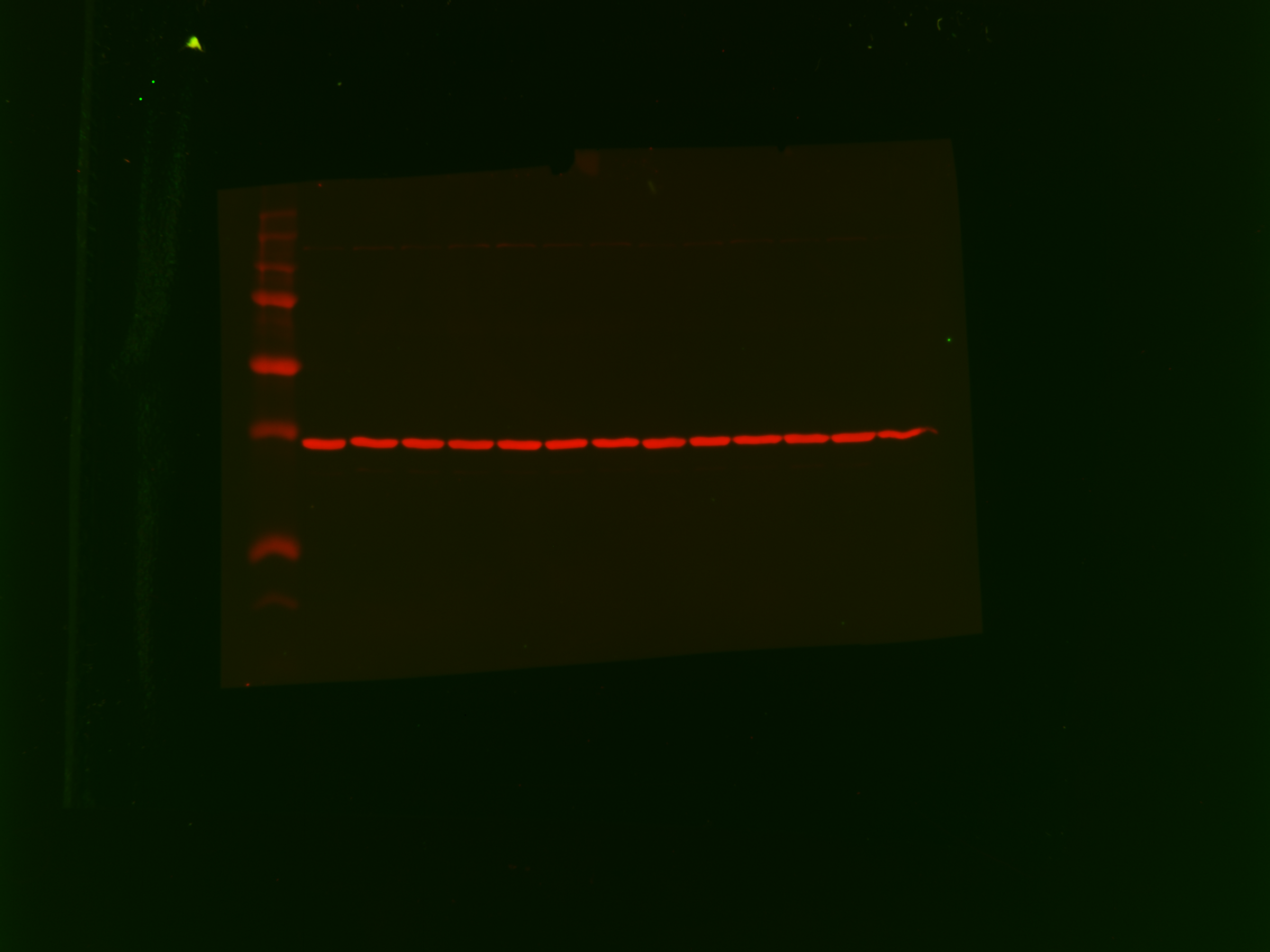

Supplement: Supplementary file 6 — Source data [file 41467_2025_64744_MOESM6_ESM.zip › 2C Membrane1 EGR1 NIR.tif]

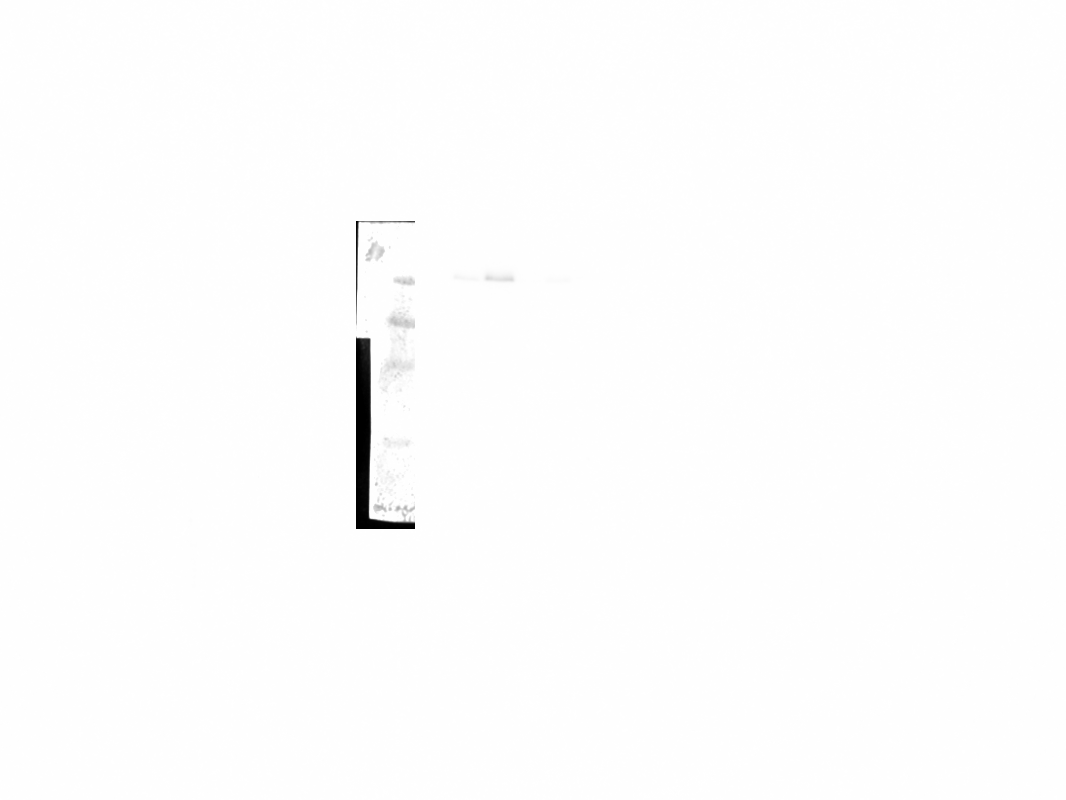

Supplement: Supplementary file 6 — Source data [file 41467_2025_64744_MOESM6_ESM.zip › 2C Membrane2 EGR1 Chemi Normal.tif]
